# Supplementary material for: India Hypertension Control Initiative: Blood Pressure Control Using Drug and Dose-Specific Standard Treatment Protocol at Scale in Punjab and Maharashtra, India, 2022
Source: Glob Heart. 2024 Mar 19;19(1):30. doi: 10.5334/gh.1305 (PMC10959138; doi:10.5334/gh.1305)
Supplement: Supplementary Files. — Supplementary Figures 1 and 2 and Tables 1 to 3. [file gh-19-1-1305-s1.pdf]

Supplementary Figure 1

Click here to access/download;Supplementary Files;Supplementary figure 1.png

Punjab

Hypertension Protocol

Measure blood pressure of **all adults** over 18 years

GOVT. PUNJAB

High BP: **SBP ≥ 140** or **DBP ≥ 90** mmHg

Step 1 — If BP is high:*
Prescribe Amlodipine 5mg

Step 2 — After 30 days# measure BP again. If still high:
Increase to Amlodipine 10mg

Step 3 — After 30 days# measure BP again. If still high:
Add Telmisartan 40mg

Step 4 — After 30 days# measure BP again. If still high:
Increase to Telmisartan 80mg

Step 5 — After 30 days# measure BP again. If still high:
Add Chlorthalidone 12.5mg**

Step 6 — After 30 days# measure BP again. If still high:
Increase to Chlorthalidone 25mg**

... — After 30 days measure BP again. If still high:
Check if the patient has been taking medications regularly and correctly. If yes, refer to a specialist.

- * If SBP ≥ 180 or DBP ≥ 110, refer patient to a specialist after starting treatment.
  If SBP 160-179 or DBP 100-109, start treatment on the same day.
  If SBP 140-159 or DBP 90-99, check on a different day and if still elevated, start treatment.
- \# Dose of anti-hypertension medications can be titrated at 15 days frequency if required.
- ** Hydrochlorothiazide can be used if Chlorthalidone is not available (25 mg starting dose, 50 mg intensification dose).

Pregnant women and women who may become pregnant

▲ DO NOT give Telmisartan or Chlorthalidone.
- Statins, ACE inhibitors, angiotensin receptor blockers (ARBs), and thiazide/thiazide-like diuretics should not be given to pregnant women or to women of childbearing age not on effective contraception.
- Calcium channel blocker (CCB) can be used. If not controlled with intensification dose, refer to a specialist.

Diabetic patients
- Treat diabetes according to protocol.
- Aim for a BP target of < 140/90 mmHg.

Heart attack in last 3 years
- Add beta blocker to Amlodipine with initial treatment.

Heart attack or stroke, ever
- Begin low-dose aspirin (75mg) and statin.

People with high CVD risk
- Consider aspirin and statin.

Chronic kidney disease
- ACEI or ARB preferred if close clinical and biochemical monitoring is possible.

Lifestyle advice for all patients

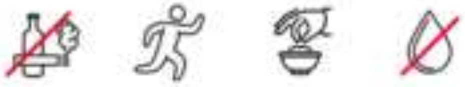

Avoid tobacco and alcohol | Exercise 2.5 hr/week | Reduce salt, under 1 tsp/day | Eat less fried foods

Eat 5 servings of fruits and vegetables per day.

Avoid papads, chips, chutneys, dips, and pickles.

Use healthy oils: E.g. sunflower, mustard, or groundnut.

Limit consumption of foods containing high amounts of saturated fats.

Reduce weight if overweight.

Reduce fat intake by changing how you cook:
- Remove the fatty part of meat
- Use vegetable oil
- Boil, steam, or bake instead of fry
- Limit reuse of oil for frying

Avoid processed foods containing trans fats.

Avoid added sugar.

IHCI India Hypertension Control Initiative

- Dispense drugs for 30 days and give appointment after 4 weeks
- Medications should be taken at the same time each day

Supplementary Figure 2

Click here to access/download;Supplementary Files;Supplementary figure 2.png

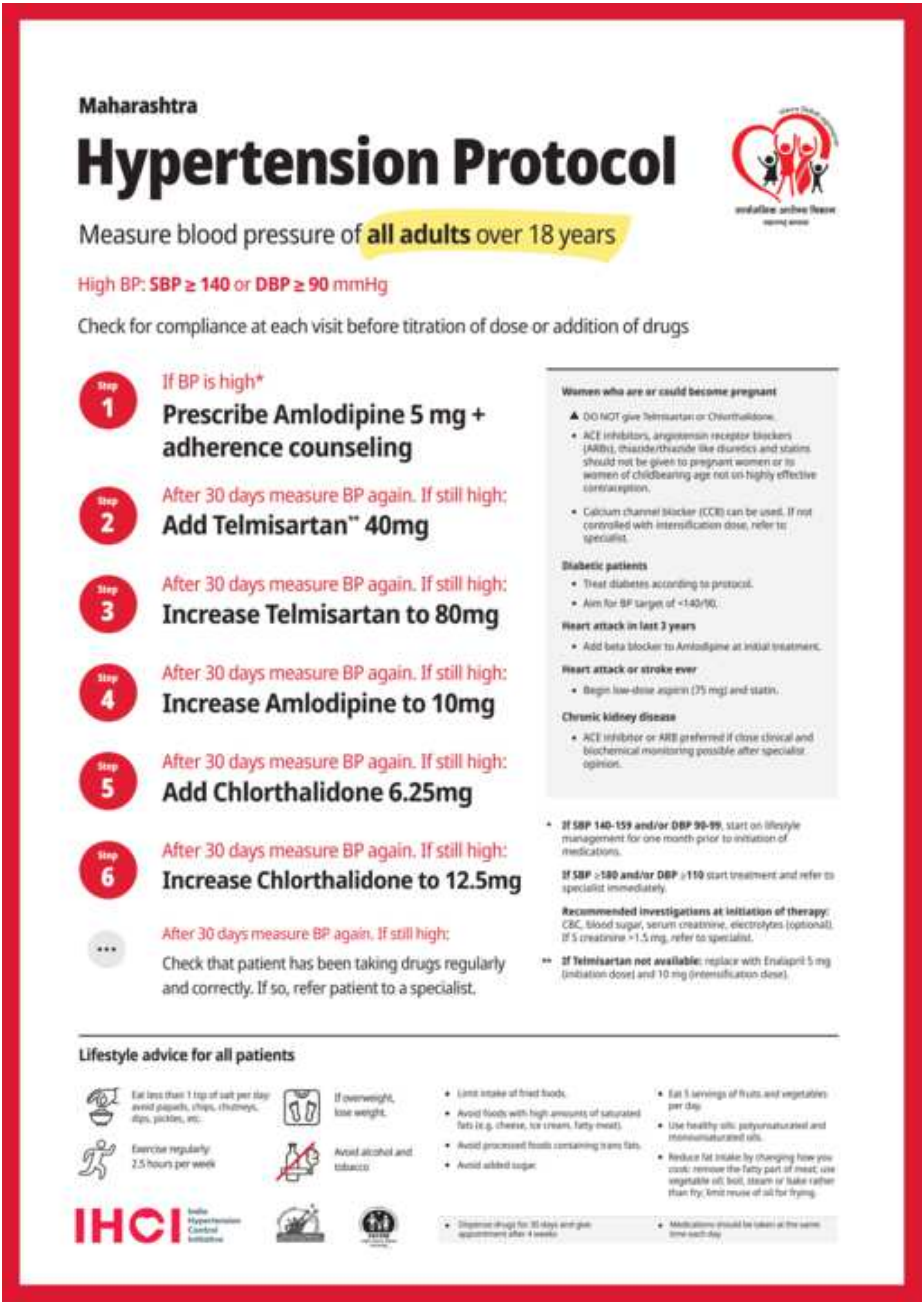

Maharashtra

Hypertension Protocol

Measure blood pressure of **all adults** over 18 years

High BP: **SBP ≥ 140** or **DBP ≥ 90** mmHg

Check for compliance at each visit before titration of dose or addition of drugs

Step 1 — If BP is high*

Prescribe Amlodipine 5 mg + adherence counseling

Step 2 — After 30 days measure BP again. If still high:

Add Telmisartan 40mg**

Step 3 — After 30 days measure BP again. If still high:

Increase Telmisartan to 80mg

Step 4 — After 30 days measure BP again. If still high:

Increase Amlodipine to 10mg

Step 5 — After 30 days measure BP again. If still high:

Add Chlorthalidone 6.25mg

Step 6 — After 30 days measure BP again. If still high:

Increase Chlorthalidone to 12.5mg

... After 30 days measure BP again. If still high:

Check that patient has been taking drugs regularly and correctly. If so, refer patient to a specialist.

Women who are or could become pregnant

- ▲ DO NOT give Telmisartan or Chlorthalidone.
- ACE inhibitors, angiotensin receptor blockers (ARBs), thiazide/thiazide like diuretics and statins should not be given to pregnant women or to women of childbearing age not on highly effective contraception.
- Calcium channel blocker (CCB) can be used. If not controlled with intensification dose, refer to specialist.

Diabetic patients

- Treat diabetes according to protocol.
- Aim for BP target of <140/90.

Heart attack in last 3 years

- Add beta blocker to Amlodipine at initial treatment.

Heart attack or stroke ever

- Begin low-dose aspirin (75 mg) and statin.

Chronic kidney disease

- ACE inhibitor or ARB preferred if close clinical and biochemical monitoring possible after specialist opinion.

* **If SBP 140-159 and/or DBP 90-99**, start on lifestyle management for one month prior to initiation of medications.

If SBP ≥180 and/or DBP ≥110 start treatment and refer to specialist immediately.

Recommended investigations at initiation of therapy: CBC, blood sugar, serum creatinine, electrolytes (optional). If S creatinine >1.5 mg, refer to specialist.

** **If Telmisartan not available**: replace with Enalapril 5 mg (initiation dose) and 10 mg (intensification dose).

Lifestyle advice for all patients

Eat less than 1 tsp of salt per day avoid papads, chips, chutneys, dips, pickles, etc.

If overweight, lose weight.

Exercise regularly 2.5 hours per week

Avoid alcohol and tobacco

- Limit intake of fried foods.
- Avoid foods with high amounts of saturated fats (e.g. cheese, ice cream, fatty meat).
- Avoid processed foods containing trans fats.
- Avoid added sugar.
- Eat 5 servings of fruits and vegetables per day.
- Use healthy oils: polyunsaturated and monounsaturated oils.
- Reduce fat intake by changing how you cook: remove the fatty part of meat; use vegetable oil; boil, steam or bake rather than fry; limit reuse of oil for frying.

IHCI India Hypertension Control Initiative

- Dispense drugs for 30 days and give appointment after 4 weeks
- Medications should be taken at the same time each day

India Hypertension Control Initiative- Blood pressure control using drug and dose-specific standard treatment protocol at scale in Punjab and Maharashtra, India, 2022

Supplementary Table 1. Characteristics of individuals registered under IHCI but excluded* from the analysis

Characteristic	Category	Punjab (N=27,688)		Maharashtra (N=94,229)		Total (N=121,917)	
		n	%	n	%	n	%
Age groups (in years)	<30	483	0.4	1,053	0.3	1,536	0.3
	30-49	20,300	18.6	53,740	16.0	74,040	16.7
	50-69	65,840	60.4	2,04,197	60.9	2,70,037	60.8
	≥70	22,371	20.5	76,521	22.8	98,892	22.2
Gender	Male	39,757	36.5	1,45,950	43.5	1,85,707	41.8
	Female	69,237	63.5	1,89,561	56.5	2,58,798	58.2
Diabetes	Yes	28,309	26.0	1,10,928	33.1	1,39,237	31.3
	No	80,685	74.0	2,24,583	66.9	3,05,268	68.7
Facility type	SDH/DH	16,814	15.4	62,072	18.5	78,886	17.7
	CHC	24,624	22.6	13,804	4.1	38,428	8.6
	PHC	15,256	14.0	1,36,613	40.7	1,51,869	34.2
	HWC	52,300	48.0	1,23,022	36.7	1,75,322	39.4
Baseline BP grades	Controlled BP	3,447	12.4	1,13,543	33.8	1,16,990	32.2
	Grade I	14,290	51.6	1,16,923	34.8	1,31,213	36.1
	Grade II	9,951	35.9	1,05,045	31.3	1,14,996	31.7
Baseline Systolic BP	Mean (SD)	153.5 (19.7)		142.7 (20.0)		145.8 (18.0)	
Baseline Diastolic BP	Mean (SD)	89.9 (11.5)		85.6 (11.8)		87.1 (11.1)	

*Exclusions - Taking drugs other than Amlodipine 5mg or lost-to-follow-up or taking drugs other than those included in the IHCI regimen
DH – District Hospital; SDH – Sub-district Hospital; CHC – Community Health Centre; PHC – Primary Health Centre; HWC – Health and Wellness Centre

Supplementary Table 2. Comparison of characteristics of individuals included & excluded* from the analysis

Characteristics	Category	Included, N=159,292		Excluded, N=121,917	
		n	%	n	%
Age groups (in years)	<30	392	0.2	424	0.3
	30-49	25,515	16.0	20,147	16.5
	50-69	95,620	60.0	72,771	59.7
	≥70	37,765	23.7	28,575	23.4
Gender	Male	58,760	36.9	1,55,279	42.8
	Female	1,00,532	63.1	2,07,920	57.2
Diabetes	Yes	12,038	7.6	1,16,647	32.1
	No	1,47,254	92.4	2,46,552	67.9
Facility type	SDH / DH	13,147	8.3	65,525	18.0
	CHC	9,111	5.7	20,466	5.6
	PHC	53,012	33.3	1,41,056	38.8
	HWC	84,022	52.7	1,36,152	37.5
Baseline BP grades	Controlled BP	45,151	28.3	1,16,990	32.2
	Grade I	73,152	45.9	1,31,213	36.1
	Grade II	40,989	25.7	1,14,996	31.7
Baseline Systolic BP	Mean (SD)	144.7 (17.3)		145.8 (18.0)	
Baseline Diastolic BP	Mean (SD)	86.9 (10.7)		87.1 (11.1)	

*Exclusions - Lost-to-follow-up or taking drugs other than those included in the IHCI regimen during follow-up

DH – District Hospital; SDH – Sub-district Hospital; CHC – Community Health Centre; PHC – Primary Health Centre; HWC – Health and Wellness Centre

Supplementary Table 3: Baseline and follow-up blood pressure among individuals started on Amlo 5 mg on registration up to Dec 2021 and had at least one follow-up visit between 01 Jan 2022 to 31 Mar 2022 (excluding missing BP values at follow-up visit)

Systolic BP		Punjab				Maharashtra			
Characteristic	Category	N	Mean SBP at baseline ± SD	Mean SBP at recent follow up ± SD	Mean Difference SBP (95% CI)*	N	Mean SBP at baseline ± SD	Mean SBP at recent follow up ± SD	Mean Difference SBP (95% CI)*
Age Category (years)	<30	144	147 ± 13	129 ± 11	18 (21 - 15)	240	144 ± 16	130 ± 12	14 (16 - 12)
	30-49	7,058	147 ± 14	131 ± 11	16 (16 - 15)	18,003	145 ± 17	129 ± 12	16 (16 - 15)
	50-69	19,324	148 ± 15	132 ± 12	16 (16 - 16)	74,385	144 ± 18	129 ± 12	15 (15 - 15)
	≥70	6,439	149 ± 16	132 ± 13	17 (18 - 17)	30,569	144 ± 19	129 ± 12	15 (15 - 14)
Gender	Male	10,108	149 ± 15	132 ± 12	17 (17 - 16)	47,560	146 ± 18	130 ± 12	16 (16 - 15)
	Female	22,857	147 ± 15	131 ± 12	16 (16 - 16)	75,637	143 ± 18	129 ± 12	14 (14 - 14)
Diabetes	Yes	5,235	148 ± 15	132 ± 13	16 (16 - 15)	6,535	144 ± 18	130 ± 13	14 (14 - 13)
	No	27,730	148 ± 15	132 ± 12	16 (16 - 16)	1,16,662	144 ± 18	129 ± 12	15 (15 - 15)
Facility type	SDH / DH	1,987	153 ± 17	135 ± 16	18 (19 - 17)	10,993	144 ± 19	131 ± 15	12 (13 - 12)
	CHC	4,587	151 ± 16	134 ± 14	17 (18 - 17)	4,421	145 ± 19	131 ± 14	14 (15 - 13)
	PHC	4,304	149 ± 16	133 ± 13	16 (17 - 16)	47,586	145 ± 18	129 ± 12	15 (15 - 15)
	HWC	22,087	147 ± 14	131 ± 11	16 (16 - 16)	60,197	143 ± 17	128 ± 11	15 (15 - 15)
	Overall	**32,965**	**148 ± 15**	**132 ± 12**	**16 (16 - 16)**	**1,23,197**	**144 ± 18**	**129 ± 12**	**15 (15 - 15)**
Diastolic BP		Punjab				Maharashtra			
Characteristic	Category	N	Mean DBP at baseline ± SD	Mean DBP at recent follow up ± SD	Mean Difference DBP (95% CI)*	N	Mean DBP at baseline ± SD	Mean DBP at recent follow up ± SD	Mean Difference DBP (95% CI)*
Age Category (years)	<30	144	90 ± 10	83 ± 8	7 (9 - 6)	240	89 ± 12	81 ± 9	8 (10 - 7)
	30-49	7,058	90 ± 9	81 ± 7	9 (9 - 8)	18,003	90 ± 11	81 ± 8	10 (10 - 10)
	50-69	19,324	88 ± 10	81 ± 7	7 (7 - 7)	74,385	87 ± 11	80 ± 8	7 (7 - 7)
	≥70	6,439	86 ± 10	80 ± 8	6 (6 - 6)	30,569	84 ± 11	79 ± 8	6 (6 - 5)

**Gender**	Male	10,108	89 ± 10	81 ± 7	8 (8 - 7)	47,560	88 ± 11	80 ± 8	8 (8 - 8)
	Female	22,857	88 ± 9	81 ± 7	7 (7 - 7)	75,637	86 ± 11	79 ± 8	7 (7 - 6)
**Diabetes**	Yes	5,235	88 ± 10	81 ± 8	7 (7 - 7)	6,535	86 ± 11	80 ± 8	6 (6 - 6)
	No	27,730	88 ± 10	81 ± 7	7 (7 - 7)	1,16,662	87 ± 11	80 ± 8	7 (7 - 7)
**Facility type**	SDH / DH	1,987	88 ± 11	81 ± 9	7 (8 - 6)	10,993	86 ± 11	80 ± 9	6 (7 - 6)
	CHC	4,587	88 ± 10	80 ± 8	8 (8 - 7)	4,421	86 ± 11	80 ± 9	6 (7 - 6)
	PHC	4,304	88 ± 10	82 ± 7	7 (7 - 6)	47,586	87 ± 11	80 ± 8	7 (7 - 7)
	HWC	22,087	88 ± 9	81 ± 7	7 (7 - 7)	60,197	87 ± 11	79 ± 7	7 (7 - 7)
	**Overall**	**32,965**	88 ± 10	**81 ± 7**	**7 (7 - 7)**	**1,23,197**	**87 ± 11**	**80 ± 8**	**7 (7 - 7)**

* Paired t test (1-tail p-value) <0.001

STROBE Statement

India Hypertension Control Initiative- Blood pressure control using drug and dose-specific standard treatment protocol at scale in Punjab and Maharashtra, India, 2022

	Item No	Recommendation	Page No
**Title and abstract**	1	(*a*) Indicate the study's design with a commonly used term in the title or the abstract	NA (secondary data analysis of program data)
		(*b*) Provide in the abstract an informative and balanced summary of what was done and what was found	1
**Introduction**			
Background/rationale	2	Explain the scientific background and rationale for the investigation being reported	2
Objectives	3	State-specific objectives, including any prespecified hypotheses	3
**Methods**			
Study design	4	Present key elements of study design early in the paper	3
Setting	5	Describe the setting, locations, and relevant dates, including periods of recruitment, exposure, follow-up, and data collection	3
Participants	6	(*a*) Give the eligibility criteria, and the sources and methods of selection of participants	6, 7
Variables	7	Clearly define all outcomes, exposures, predictors, potential confounders, and effect modifiers. Give diagnostic criteria, if applicable	6, 7
Data sources/ measurement	8*	For each variable of interest, give sources of data and details of methods of assessment (measurement). Describe comparability of assessment methods if there is more than one group	5
Bias	9	Describe any efforts to address potential sources of bias	6
Study size	10	Explain how the study size was arrived at	6, 7
Quantitative variables	11	Explain how quantitative variables were handled in the analyses. If applicable, describe which groupings were chosen and why	6, 7
Statistical methods	12	(*a*) Describe all statistical methods, including those used to control for confounding	7
		(*b*) Describe any methods used to examine subgroups and interactions	7
		(*c*) Explain how missing data were addressed	7
		(*d*) If applicable, describe analytical methods taking account of sampling strategy	NA
		(*e*) Describe any sensitivity analyses	NA

**Results**			
Participants	13*	(a) Report numbers of individuals at each stage of study—eg numbers potentially eligible, examined for eligibility, confirmed eligible, included in the study, completing follow-up, and analysed	NA
		(b) Give reasons for non-participation at each stage	NA
		(c) Consider use of a flow diagram	NA
Descriptive data	14*	(a) Give characteristics of study participants (eg demographic, clinical, social) and information on exposures and potential confounders	7
		(b) Indicate number of participants with missing data for each variable of interest	9
Outcome data	15*	Report numbers of outcome events or summary measures	10
Main results	16	(*a*) Give unadjusted estimates and, if applicable, confounder-adjusted estimates and their precision (eg, 95% confidence interval). Make clear which confounders were adjusted for and why they were included	NA
		(*b*) Report category boundaries when continuous variables were categorized	8, 10
		(*c*) If relevant, consider translating estimates of relative risk into absolute risk for a meaningful time period	NA
Other analyses	17	Report other analyses done—eg analyses of subgroups and interactions, and sensitivity analyses	8
**Discussion**			
Key results	18	Summarise key results with reference to study objectives	11
Limitations	19	Discuss limitations of the study, taking into account sources of potential bias or imprecision. Discuss both direction and magnitude of any potential bias	14
Interpretation	20	Give a cautious overall interpretation of results considering objectives, limitations, multiplicity of analyses, results from similar studies, and other relevant evidence	15
Generalisability	21	Discuss the generalisability (external validity) of the study results	15
**Other information**			
Funding	22	Give the source of funding and the role of the funders for the present study and, if applicable, for the original study on which the present article is based	16

*Give information separately for exposed and unexposed groups.

Note: An Explanation and Elaboration article discusses each checklist item and gives methodological background and published examples of transparent reporting. The STROBE checklist is best used in

conjunction with this article (freely available on the Web sites of PLoS Medicine at http://www.plosmedicine.org/, Annals of Internal Medicine at http://www.annals.org/, and Epidemiology at http://www.epidem.com/). Information on the STROBE Initiative is available at www.strobe-statement.org.
